# Application of prothrombin complex concentrate for reversal of direct oral anticoagulants in clinical practice: indications, patient characteristics and clinical outcomes compared to reversal of vitamin K antagonists

**DOI:** 10.1186/s13049-019-0625-3

**Published:** 2019-04-23

**Authors:** Martin Müller, Jonathan Eastline, Michael Nagler, Aristomenis K. Exadaktylos, Thomas C. Sauter

**Affiliations:** 1 Department of Emergency Medicine, Inselspital, Bern University Hospital, Bern University, Bern, Switzerland; 2 Department of Health Economics and Clinical Epidemiology, Cologne University Hospital, Cologne, Germany; 3 University Institute of Clinical Chemistry, Inselspital Bern University Hospital, and University of Bern, Bern, Switzerland; 4 Charité Medical School Berlin, Medical Skills Lab, Charité, Berlin, Germany

**Keywords:** Anticoagulants, Antidote, Bleeding, Direct oral anticoagulants, Vitamin K antagonist

## Abstract

**Background:**

Prothrombin complex concentrate (PCC) is widely used to reverse the action of direct oral anticoagulants (DOACs) in accordance with current guidelines and because of a lack of specific reversal agents. Indications, clinical characteristics and patient outcomes of patients might differ in comparison to reversal of vitamin K antagonists where reversal with PCC is well established.

**Methods:**

Our cohort study explores patient characteristics, indications and clinical outcomes for reversal of all DOAC patients receiving PCC at our university emergency department from 01.06.2012 to 01.07.2017, in comparison with patients on VKA.

**Results:**

Out of 199,982 consultations, we studied 346 patients who were given PCC for reversal of either DOAC (*n* = 74) or VKA (*n* = 272). The most common reason for treatment was acute bleeding; in 86.7% of both groups. 37.3% of bleeding was traumatic (*p* = 0.666). The most frequent bleeding location was intracranial (61.6%, *p* = 0.881). Gastrointestinal bleeding was more often found in the DOAC group (18.9% vs. 8.8%, *p* = 0.014). More erythrocyte concentrates (ECs) were given to DOAC patients with blood transfusion (*p* = 0.014). Tranexamic acid was used more often in DOAC patients than in VKA patients (28.4% vs. 7.4%, *p* < 0.001). No significant group differences were found for the following patient outcomes: in-hospital mortality, ICU stay, and length of stay at the ICU or in hospital.

**Conclusion:**

In DOAC treated patients, PCC was applied more often because of gastrointestinal bleeding and patients received higher numbers of ECs as well as tranexamic acid. No differences were observed with regard to important clinical outcomes.

## Background

The main drawback when dealing with direct oral anticoagulants (DOACs) is the risk of bleeding. Simply stopping DOACs might be adequate for some clinical situations because of the short half-life of these drugs [1]. Nevertheless, anticoagulant activity must be rapidly reversed for life-threatening bleeding, emergency surgery or procedures. In contrast to dabigatran – where the specific reversal agent idarucizumab is available [2] –, no specific reversal agent for the factor Xa inhibitors apixaban, edoxaban and rivaroxaban has yet been approved in most countries. In cases of life-threatening and uncontrollable bleeding, adexanet alfa has recently been approved by the FDA for the reversal of apixaban and rivaroxaban but not edoxaban [3]. Clinical experience with this new reversal agent is still sparse and practical real-life indications are yet to be established [4]. Most clinicians currently use prothrombin complex concentrate (PCC) for reversal of factor Xa inhibitors, despite the lack of prospective evidence in accordance with current recommendations and guidelines [5–7]. Prothrombin complex concentrate might counteract the anti-Xa effect of rivaroxaban by restoring thrombin generation [8]. A recent multinational survey has identified large gaps in the knowledge of the management of DOAC-associated bleeding [9].

In contrast to DOAC reversal, PCC is well established as the reversal agent for VKA because of its rapid onset of action, small volume load and the low risk of transfusion reactions [5, 6].

While the prevalence of DOACs is growing and specific reversal agents are coming on the market, there is still little evidence or clinical experience with DOAC reversal. Therefore, further insights into the practice and outcome of DOAC reversal are needed.

Thus, our study aims to compare the characteristics of patients in the following two groups: those receiving PCC for non-specific reversal of DOAC and those receiving PCC for reversal of VKA. We also compared the indications for reversal in the two patient groups, together with the clinical ED outcomes (blood products given, ICU/IMC admission, length of stay (ICU/IMC and in-hospital), and in-hospital mortality).

## Methods

### Study design and setting

This retrospective cohort study was set in the adult ED of Bern University Hospital (Inselspital), Switzerland, a self-contained, interdisciplinary department, treating patients aged 18 years and older. The adult ED is a level 1 centre. Over 40,000 consultations are annually registered and treated at the ED.

### Inclusion criteria

The analysis included all patients on DOAC or VKA who received PCC - irrespective of the cause – and were admitted from 01.06.2012 to 01.07.2017 to our ED.

Our hospital follows a specific published guideline for the reversal of oral anticoagulants in the case of acute bleeding incidences or urgent procedures [5]. In our study population, the indication for the use of PCC in the individual case was provided by the attending physician. In our hospital, only four-factor PCC is used and therefore included in this study.

### Exclusion criteria

The following exclusion criteria were used:

- No documentation of the application of PCC
- No oral anticoagulation medication documented in the medical report or insufficient information to determine anticoagulation status

### Study outcomes

Study outcomes were in-hospital mortality and procedural outcomes (e.g. hospitalisation, length of hospital stay [hours], the need for ICU admission during hospitalisation and length of ICU [hours]), as well as the need for and amount of blood products given.

### Data collection and extraction

All medical reports, including patient history and diagnosis, clinical findings, medication given at the ED and on arrival, as well as procedure in and after the ED are electronically recorded for every patient and stored in the computerised ED patient database (E-Care, ED 2.1.3.0, Turnhout, Belgium).

Firstly, a broad keyword search was performed through all medical reports between 01.06.2012 to 01.07.2017 - containing all the substance classes and brand names of oral anticoagulants approved in Switzerland (substance classes: rivaroxaban, dabigatran, apixaban, and edoxaban, phenprocoumon, warfarin, and acenocoumarol), combined with the logic operator “OR”.

The application of medication, especially PCC, is thoroughly documented in the database. Secondly, we excluded all results of the keyword search that did not receive PCC – the application of PCC is electronically stored in the system. Thirdly, manual screening (done by JE) was performed through the medical report of the remaining consultations, excluding all consultations without current intake of an oral anticoagulation (e.g. paused medication), giving the final study population. Variables describing the patient’s anticoagulation, the patient and bleeding characteristics, were extracted from the medical report (done by JE). All investigated outcomes were obtained from the patient database.

### Exposure

The exposure was to all sorts of Swiss medic-approved direct oral anticoagulation drugs. We assigned the direct oral anticoagulants (rivaroxaban, dabigatran, apixaban, and edoxaban) to the DOAC group. For comparison, we used the VKA group, comprising all patients under active treatment with phenprocoumon, warfarin, or acenocoumarol.

### Data analysis

The statistical analysis was performed with Stata® 13.1 (StataCorp, The College Station, Texas, USA). The study compared patients on DOAC or VKA, which were reversed by PCC. Categorical variables were expressed as absolute numbers, accompanied by relative numbers. Continuous variables were expressed as medians with interquartile ranges. The two groups of patients (DOAC or VKA) were compared with respect to the reported clinical ED outcomes (in-hospital mortality, hospitalisation, length of hospital stay [hours], ICU admission, and length of ICU stay [hours]). Fisher’s exact tests were used to compare categorical variables and the Wilcoxon rank sum test to compare continuous variables. A *p*-value of < 0.05 was defined as significant.

### Ethics

The present study is registered with the ethics committee of Canton Bern, Switzerland (073/2015). Because of the use of coded routine care patient data, no informed consent is needed according to Swiss law.

## Results

**Fig. 1:**
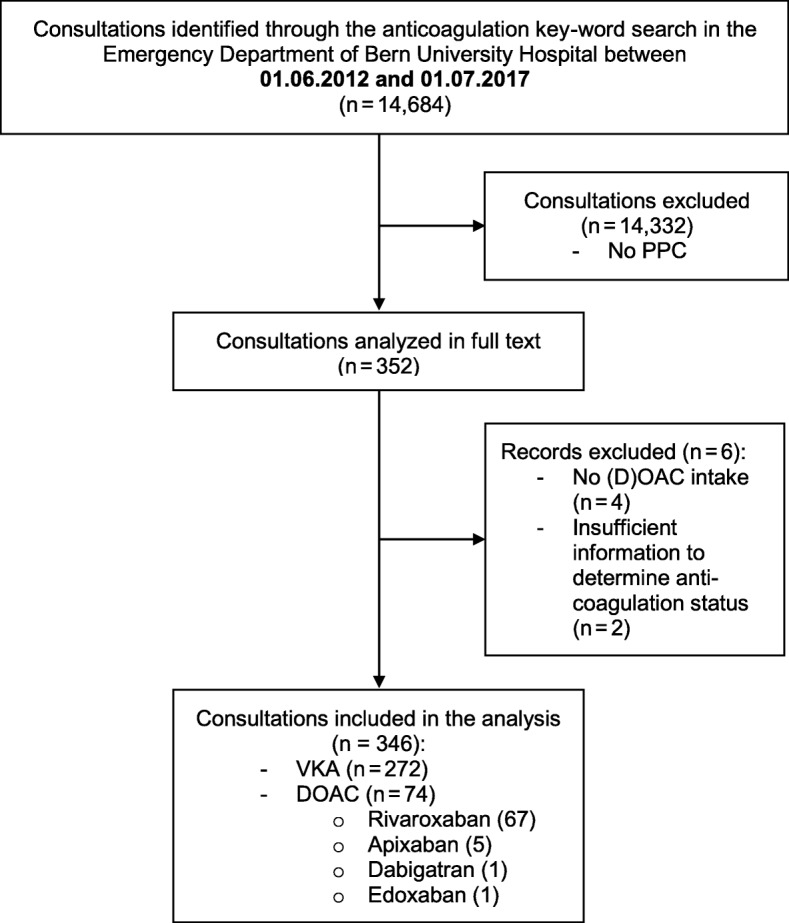
Abbreviations: DOAC, direct oral anticoagulant; VKA, Vitamin K antagonists. (D)OAC, (direct) oral anticoagulant; PPC, prothrombin complex concentrate

In total, 14,684 of 199,982 consultations (7.2%) were identified through the anticoagulation key-word search over the five-year study period (Fig. 1).

PPC was not administered in 14,332 consultations, so 352 consultations were analysed in full text. After manually screening the medications, six consultations had to be excluded as no current DOAC intake was documented. Thus, the analysis included 346 consultations that received PCC for reversal of DOAC or VKA medication - divided into 272 VKA and 74 DOAC patients. Most of the patients in the DOAC group were on rivaroxaban (90.5%, *n* = 67). Five patients (6.7%) took apixaban, and one patient each dabigatran or edoxaban.

### Patient characteristics

The median age was 77 (IQR 69–82) years in the DOAC group and 79 (72–84) years in the VKA group (*p* = 0.064). Fifty-seven percent of the patients were males, without a significant group difference (*p* = 0.518).

Apart from a diagnosis of carcinoma (DOAC 21.6%, VKA: 7.7%, *p* = 0.001), there were no significant differences between the two anticoagulation groups in regard to comorbidities (Tables 1 and 2).

**Table 1:** Patient characteristics

	Total (*n* = 346)		DOAC (*n* = 74)		Vitamin K antagonist (*n* = 272)		p
Age, [median (IQR)]	78.0	(70–84)	77.0	(69–82)	79.0	(72–84)	0.064
Gender (male)	199	(57.5)	45	(60.8)	154	(56.6)	0.518
Comorbidities							
Hypertension	146	(42.2)	30	(40.5)	116	(42.6)	0.745
Diabetes	63	(18.2)	10	(13.5)	53	(19.5)	0.238
Peripheral arterial disease	26	(7.5)	6	(8.1)	20	(7.4)	0.827
Hypertensive cardiopathy	75	(21.7)	17	(23.0)	58	(21.3)	0.760
Coronary heart disease	91	(26.3)	14	(18.9)	77	(28.3)	0.104
Valvular cardiopathy	75	(21.7)	15	(20.3)	60	(22.1)	0.741
Chronic kidney disease	99	(28.6)	25	(33.8)	74	(27.2)	0.267
Liver insufficiency	6	(1.7)	2	(2.7)	4	(1.5)	0.472
COPD	21	(6.1)	6	(8.1)	15	(5.5)	0.407
Venous thromboembolism	61	(17.6)	11	(14.9)	50	(18.4)	0.481
Aortic disease	15	(4.3)	3	(4.1)	12	(4.4)	0.893
Stroke	48	(13.9)	9	(12.2)	39	(14.3)	0.631
Carcinoma	37	(10.7)	16	(21.6)	21	(7.7)	0.001
Coagulopathy	13	(3.8)	1	(1.4)	12	(4.4)	0.220

*Abbreviation: DOAC* direct oral anticoagulants, *COPD* chronic obstructive pulmonary disease

**Table 2:** Anticoagulation characteristics

	Total (*n* = 346)		DOAC (*n* = 74)		Vitamin K antagonist (*n* = 272)		p
Anticoagulation indication							
Not specified	27	(7.8)	10	(13.5)	17	(6.2)	
Atrial fibrillation	223	(64.5)	48	(64.9)	175	(64.3)	
Thromboembolic event	74	(21.4)	13	(17.6)	61	(22.4)	
Mechanical heart-valve	17	(4.9)	1	(1.4)	16	(5.9)	
Coagulopathy	5	(1.4)	2	(2.7)	3	(1.1)	0.090
Additional platelet aggregation inhibitor therapy							
None	285	(82.4)	62	(83.8)	223	(82.0)	
Acetylsalicylic acid	44	(12.7)	9	(12.2)	35	(12.9)	
Clopidogrel	13	(3.8)	1	(1.4)	12	(4.4)	
Acetylsalicylic acid + Clopidogrel	4	(1.2)	2	(2.7)	2	(0.7)	0.328
Additional heparin	7	(2.0)	1	(1.4)	6	(2.2)	0.643

*Abbreviation: DOAC* direct oral anticoagulants

The most common indication for anticoagulation was atrial fibrillation in both groups (64.5%). There was no significant difference between the anticoagulation indication (*p* = 0.090), additional platelet therapy (*p* = 0.328), or additional heparin therapy (*p* = 0.643), see Table 2. In our population, bleeding events were not associated with additional platelet aggregation inhibitor therapy (*p* = 0.964).

### Consultation characteristics and indications for reversal

An acute life**-**threatening triage was coded significantly more often (*p* = 0.024) in the DOAC group (51.4%) than in the VKA group (31.2%), whereas less urgent triage was uncommon in both groups (DOAC: 0.0% vs. VKA: 1.5%).

Haemoglobin, platelets, and creatinine did not differ significantly between the groups (*p* = 0.094, 0.432, 0.832). The median INR was 1.2 (IQR 1.1–1.4) in the DOAC group and 2.3 (1.6–3.1) in the VKA group (*p* < 0.001).

In both groups, the most common indication for reversal was bleeding (DOAC: 93.2% vs. VKA: 84.9%); other indications were endoscopy (total: 2.9%) or invasive procedures e.g. drainage need or centesis (total: 5.2%) and emergency operation (total: 5.2%), without a significant group difference (*p* = 0.245). There was no significant difference (*p* = 0.666) between the proportion of trauma consultations (DOAC: 35.1% vs. VKA: 37.9%), see Table 3.

**Table 3:** Consultation characteristics and indications for reversal

	Total (*n* = 346)		DOAC (*n* = 74)		Vitamin K antagonist (*n* = 272)		p
Triage							
Acute life threating problem	123	(35.5)	38	(51.4)	85	(31.2)	
High urgency	142	(41.0)	25	(33.8)	117	(43.0)	
Urgency	70	(20.2)	10	(13.5)	60	(22.1)	
Less urgency	4	(1.2)	0	(0.0)	4	(1.5)	
Missing	7	(2.0)	1	(1.4)	6	(2.2)	0.024
Laboratory values^a^							
Haemoglobin (g/l)	122	(105–136)	117.5	(97–135)	122	(107–136)	0.094
Creatinine (μmol/l)	90	(71–123)	85.0	(71–118)	91	(71–125)	0.432
Platelets (G/l)	209	(168–262)	199.5	(169–271)	211	(168–254)	0.832
INR	2.0	(1.3–2.9)	1.2	(1.1–1.4)	2.3	(1.6–3.1)	< 0.001
Trauma	129	(37.3)	26	(35.1)	103	(37.9)	0.666
Outpatient discharge	2	(0.6)	1	(1.4)	1	(0.4)	0.322
Indication for reversal							
Bleeding	300	(86.7)	69	(93.2)	231	(84.9)	
Endoscopy	10	(2.9)	1	(1.4)	9	(3.3)	
Drainage, centesis, puncture	18	(5.2)	3	(4.1)	15	(5.5)	
Emergency operation	18	(5.2)	1	(1.4)	17	(6.2)	0.245

*Abbreviation: DOAC* direct oral anticoagulants, *INR* international normalised ratio

^a^Data available for 94.2, 99.1, 83.2%, or 99.1% for the values haemoglobin, creatinine, platelets, or INR

### Bleeding characteristics

As the main indication for reversal was bleeding (86.7%), we further investigated bleeding localisation in all patients (Table 4). The most common location was intracranial (61.6%), followed by gastrointestinal bleeding (11.0%) and superficial bleeding (6.4%). Apart from gastrointestinal bleeding - which was found in 18.9% of the DOAC consultations and in 8.8% of the VKA consultations (*p* = 0.014) -, no significant differences between the groups were found in regard to the bleeding location.

**Table 4:** Bleeding locations

	Total (*n* = 346)		DOAC (*n* = 74)		Vitamin K antagonist (*n* = 272)		p
Intracranial	213	(61.6)	45	(60.8)	168	(61.8)	0.881
Upper/lower gastrointestinal	38	(11.0)	24	(18.9)	14	(8.8)	0.014
Superficial	22	(6.4)	4	(5.4)	18	(6.6)	0.705
Extremity	17	(4.9)	2	(2.7)	15	(5.5)	0.321
Abdominal	13	(3.8)	2	(2.7)	11	(4.0)	0.591
Thorax	11	(3.2)	4	(5.4)	7	(2.6)	0.218
Eye	6	(1.7)	1	(1.4)	5	(1.8)	0.776
Retroperitoneal	5	(1.4)	1	(1.4)	4	(1.5)	0.939
Haematuria	3	(0.9)	1	(1.4)	2	(0.7)	0.612
Epistaxis	2	(0.6)	0	(0.0)	2	(0.7)	0.459
Oral	1	(0.3)	0	(0.0)	1	(0.4)	0.601

*Abbreviation: DOAC* direct oral anticoagulants

### Blood products and reversal

The numbers of blood products and reversals that were given in the anticoagulation groups are shown in Table 5. The number of additionally transfused erythrocyte concentrates (ECs) was 20.3% in the DOAC group and 12.1% in the VKA group (*p* = 0.073), with significant differences regarding the number of ECs given - with a higher number in the DOAC group (*p* = 0.014). The number of PCC units or platelet concentrates given did not differ between the groups (*p* = 0.346, *p* = 0.307, respectively). Tranexamic acid in addition to PCC was given significantly more often in the DOAC group (28.4% vs. 7.4%, *p* < 0.001).

**Table 5:** Blood products and reversal agents

	Total (*n* = 346)		DOAC (*n* = 74)		Vitamin K antagonist (*n* = 272)		p
PCC (units), [median (IQR)]	2100	(1800-2400)	2000	(1700–3000)	2100	(1800-2400)	0.346
ECs given	48	(13.9)	15	(20.3)	33	(12.1)	0.073
Number of ECs							
1	23	(6.7)	6	(8.2)	17	(6.2)	
2	14	(4.1)	3	(4.1)	11	(4.0)	
3	7	(2.0)	4	(5.5)	3	(1.1)	
4	2	(0.6)	2	(2.7)	0	(0.0)	
5	2	(0.6)	0	(0.0)	2	(0.7)	0.014
Platelet concentrates	5	(1.4)	2	(2.7)	3	(1.1)	0.307
Fresh frozen plasma	19	(5.5)	6	(8.1)	13	(4.8)	0.265
Tranexamic acid	41	(11.8)	21	(28.4)	20	(7.4)	< 0.001

*Abbreviation: DOAC* direct oral anticoagulants, *ECs* erythrocyte concentrates, *IQR* interquartile range, *PCC* prothrombin complex concentrate

### ED outcomes

There was a high number of ICU admissions in both groups (67.6% vs. 59.9%, *p* = 0.231). The in-hospital mortality was 9.5% in the DOAC group and 5.5% in the VKA group (*p* = 0.218). There were no significant group differences between the length of stay at the ICU (*p* = 0.391) or in the hospital (*p* = 0.756), Table 6.

**Table 6:** Procedural outcomes

	Total (*n* = 346)		DOAC (*n* = 74)		Vitamin K antagonist (*n* = 272)		p
ICU admission	213	(61.6)	50	(67.6)	163	(59.9)	0.231
LOS ICU [hours]	16.8	(0–43)	22.0	(0–58)	16.5	(0–41)	0.391
LOS hospital [hours]	120.8	(65–225)	117.6	(72–227)	122.3	(64–224)	0.756
In-hospital mortality	22	(6.4)	7	(9.5)	15	(5.5)	0.218

*Abbreviation: DOAC* direct oral anticoagulants, *ICU* intensive care unit, *LOS* length of stay

## Discussion

### Overview

The characteristics of patients who received PCC for non-specific reversal of DOAC were found to be similar to those who underwent VKA reversal. The vast majority of reversals in both OAC groups were performed because of acute bleeding; about a third were in trauma patients. The main indication for reversal in both groups was intracranial bleeding, but significantly more DOAC patients underwent reversal for gastrointestinal bleeding than with VKA patients. A higher percentage of patients with DOAC received tranexamic acid during ED treatment, mostly because of intracranial bleeding. Patients with blood transfusion on DOACs needed larger number of ECs.

### Patient and consultation characteristics receiving PCC for non-specific reversal of DOAC compared to VKA

The age of patients receiving PCC for OAC reversal did not differ significantly between the DOAC and VKA groups. Also, no significant differences were found in comparison of all comorbidities between the two groups - apart from carcinoma. The greater percentage of patients with cancer in patients with DOAC receiving reversal is interesting, given the fact that the discussion about DOAC therapy and the effectiveness of DOAC in cancer patients as well as the bleeding risk in this population is still ongoing [10]. The efficacy and safety in cancer patients in a large randomised, controlled Hokusai VTA trail could recently be shown for edoxaban [11]. Unfortunately because of the retrospective extraction of routine emergency documentation with limited data, no statement about possible reasons for DOAC prescription in those cancer patients or the timing of prescription or cancer diagnosis can be made.

Another risk factor for bleeding events is co-medication with platelet aggregation inhibitor therapy, as shown in real life as well as in phase II studies of all DOACs as well as for VKA [12–16]. In our study, a relevant number of patients were under antiplatelet co-medication but without difference between the two groups. This co-medication was not associated with bleeding events.

Patient with DOAC reversal were found to be triaged to a more acute triage category. The reason for this impression remains unclear, especially given the fact that we did not find any difference in type of bleeding or trauma. It might be hypothesised that physicians are more cautious when treating bleeding emergencies under DOAC because they might have less experience with DOAC bleeding than with the well-known VKA. This uncertainty with the management of DOAC-associated bleeding was recently described by Shaw et al., especially for emergency physicians and anaesthetists, who are also the main groups of physicians responsible for the treatment decisions in our study [9].

### Indications for reversal

The vast majority of patients in both OAC groups received reversal because of traumatic or non-traumatic acute bleeding, but only the minority for urgent procedures or surgery. The probable explanation is that non-vital interventions can be postponed until the anticoagulant effect of DOAC has disappeared or until the effect of VKA is slowly reversed with vitamin K rather than PPC. This avoids the possible prothrombotic effect of all reversal therapy. By far the most common indication for reversal was intracranial bleeding in both patient groups, as the risk benefit is absolutely clear in those patients and no other way of bleeding control is available.

Patients with DOAC therapy received reversal more often because of gastrointestinal bleeding in comparison to patients with VKA. It is known that high-dose dabigatran and rivaroxaban, the predominant DOAC in our population, as well as high-dose edoxaban, have a greater risk of gastrointestinal bleeding compared to warfarin because of local and systemic effects [17, 18]. With our study data, no statement about the absolute incidence of gastrointestinal bleeding can be made.

### Clinical ED outcomes

#### Blood products given

Our investigation showed no significant difference in the number of patients who received ECs. There is a trend for more ECs in DOAC patients, although this does not attain significance (may be due to the limited sample size). In contrast to this, in cases when ECs were given, a significantly higher number of ECs were used in the DOAC group than in the VKA group. The reason for this is unclear. It cannot simply be explained with differences in the initial Hb, which we found to be non-significantly different. The recommendations for EC transfusion given in our in-house guidelines are the same for all types of oral anticoagulants [5]. In our institution during study period, targets for EC transfusion were haemoglobin > 70 g/l and mean arterial pressure > 50 mmHg for healthy patients; haemoglobin > 90 g/l and mean arterial pressure > 60–70 mmHg for patients with traumatic brain injury or coronary artery disease.

More patients with DOAC reversal received tranexamic acid than in the VKA group, especially patients with intracranial bleeding. The application of tranexamic acid is in line with our in-house guidelines and with guidelines that generally recommend tranexamic acid together with PPC for DOAC reversal [5–7]. About half of the intracranial bleedings were non-traumatic. The recent TICH-2 study could not show any improvement in functional status for non-traumatic intracranial bleeding after the administration of tranexamic acid compared to placebo after 90 days, but could not exclude small effects in subgroups [19]. For the subgroup of patients with DOAC, the effect is still unclear, as the TICH-NOAC study (NCT02866838) investigating tranexamic acid in spontaneous intracranial bleeding patients with DOAC therapy is still ongoing.

#### Procedural outcomes

None of the procedural outcomes (ICU admission, LOS ICU or hospital overall and in-hospital mortality) that were compared in our study differed significantly between the DOAC and VKA groups. Because of the small group sizes, it is likely that our investigation is underpowered to demonstrate differences in mortality and further research is necessary.

### Implications for the future

With the upcoming reversal agent for factor Xa antagonists, adexanet alfa, a discussion about indications for reversal is warranted given the missing experiences and presumably the remarkable costs of the specific antidote. For this discussion, it is important to understand in which situations in current practice, DOAC reversal is performed, especially because in many situations stopping DOAC therapy might be enough. Other possibilities will have to be explored, such as measuring the level of DOAC medication, in order to identify patient groups that might have the highest benefit from the specific antidote. DOAC medication levels measurements are already available in many departments. In contrast to routine DOAC level measurements, for life-threatening bleeding a fast point-of-care measurement would be necessary to guide reversal. At the moment, in our setting for severe but not life-threating, DOAC levels are obtained before reversal.

### Limitations

As our investigation is limited to one single centre, the transferability to other patient populations remains unknown and warrants further investigations.

Documentation bias cannot be completely excluded in any retrospective investigation, despite careful review of all included data. Nevertheless, this bias can be assumed to be equally distributed between all patient groups and therefore will most likely not compromise the conclusion of this study.

The distribution of the different DOACs, with rivaroxaban being the DOAC with the highest prevalence in our data, reflects the DOAC distribution in our population [20]. Because of the limited prevalence of patients with dabigatran in our population, idarucizumab is used rarely in our population and is therefore not included in this study. During the study period 4 patients received idarucizumab for dabigatran reversal in our department. Our experience with idarucizumab in a patient with impaired kidney function has been previously published [21]. The general prevalence of patients on anticoagulation is also mirrored in group size of anticoagulation vs. no-anticoagulation group.

Further prospective multi-centre investigations are recommended. These should include more patients on different DOACs and ensure sufficient power for specific outcomes. Because of the limited numbers, no statement about the specific DOACs can be made and all DOACs are analysed together. DOAC levels should be measured, in order to evaluate the influence of possible medication interactions [22] or medication compliance.

## Conclusion

Age and gender of patients receiving PCC for non-specific reversal of DOAC are comparable to patients on VKA. The vast majority of reversals in both OAC groups are performed because of acute bleeding, most frequently intracranial bleeding. A third of reversals were done in trauma patients. Reversal of gastrointestinal bleeding was performed more often in DOAC than in VKA. The reason for this and the need of higher amounts of ECs in DOAC patients with blood transfusion are unexplained.

## Abbreviations

DOAC: Direct oral anticoagulant
ED: Emergency department
ICU: Intensive care unit
LOS: Length of stay
PCC: Prothrombin complex concentrate
VKA: Vitamin K antagonists
